# Protective effects of L-carnitine and homogenized testis tissue on the testis and sperm parameters of busulfan-induced infertile male rats

**Published:** 2013-09

**Authors:** Farzaneh Dehghani, Ashraf Hassanpour, Aghdas Poost-pasand, Ali Noorafshan, Saeid Karbalay-Doust

**Affiliations:** 1 *Histomorphometry and Stereology Research Centre, Shiraz University of Medical Sciences, Shiraz, Iran.*; 2 *Department of Anatomy, Shiraz University of Medical Sciences, Shiraz, Iran.*

**Keywords:** Sperm, L-carnitine, Busulfan, Testis, fertility*.*

## Abstract

**Background:** Busulfan(Bus) is a chemotherapy drug that is widely used for cancer treatment. However, administration of busulfan may cause temporary or permanent sterility in male patients. Therefore, reduction of this side is necessary.

**Objective:** evaluation of the protective effects of L-carnitine and testis homogenized tissue(THT) on sperm parameters and the testis structure after busulfan treatment.

**Materials and Methods:** Twenty rats were divided four groups. Group I (Control) received a single dose of DMSO and 1mL of distilled water (I.P.). Group II (Bus) received a single of busulfan (10 mg/kg) plus 1 ml of the distilled water(I.P.). Group III (Bus+THT) received busulfan plus 1mL of THT daily by oral gavages. Group IV (Bus+L-car) received a single dose of busulfan plus 100 mg/kg/day L-carnitine(I.P.). after 48 dayst, the Stereological technique was used for the estimating volume and diameter of testis, seminiferous tubules and interstitial tissue, flagella length, germinal epithelium height and spermatoginic cell number. Semen analysis was used for the assessment of sperm parameters.

**Results:** THT increased volume of testis (6.5%), seminiferous tubule and interstitial tissue volume (6.5%), 6.9% and 11.7% respectively), germinal epithelium height (13%), sperm count (7.5%), and decreased sperm with abnormal morphology (1%) in comparison with the L-carnitine in busulfan treated group.

**Conclusion:** It seems the use of L-carnitine and THT decreases side effects of busulfan on the male reproductive system. However, in our study, THT is more effective than L-carnitine and leads to the recovery testis structure and sperm parameters after treatment with busulfan.

This article extracted from M.Sc. thesis. (Ashraf Hassanpour)

## Introduction

Busulfan is a chemotherapy drug that is widely used for cancer treatment. However, it has many side effects on different organs such as the bladder, liver, skin, nervous system and gonads (1). Oligospermia and azoospermia have been demonstrated after the administration of busulfan (2-5). 

Therefore, a research into drugs or materials to reduce the side effects of busulfan on the male reproductive system is necessary. Previous studies have shown that melatonin and liver growth factor have protective effects on the sperm parameter and testicular damage in busulfan treated animals (6-7). L-carnetine also has some beneficial effects on the spermatogenesis, sperm maturation and sperm motility (8). L-carnitine is a small water-soluble molecule important in mammalian fat metabolism (9). It is known that L-carnitine and its derivatives have antioxidant and anti-inflammatory effects on various pathophysiological conditions (10-12). L-carnitine has been recently shown to act as an important antiapoptotic mediator (13). 

In addition, L-carnitine enhances the activity of DNA repairing enzyme poly (ADP-ribosyl) polymerase and also other related repair mechanisms (14). The use of L-carnitine and its derivatives in therapy has been proposed in recent years for the treatment of male infertility, and a number of human and animal studies have been published that indicate a possible role for application of carnitine as an antioxidant and free radical scavenger that can improve seminal fluid quality, but, however, the mechanisms by which carnitines control male fertility are not yet understood and they cause some side effects such as nausea, vomiting, stomach upset, seizures, diarrhea and heartburn    (8,15) . 

In the early 19^th^ century, the scientists attempted to extract materials from the body tissues that could be used in the treatment of some diseases. Investigations show that the applications of kidney extract were used for reducing blood pressure or inhibiting the growth of transplantable tumors by testis extract (16, 17). Testis extract contains several agents such as spermin, and especially, spermatogonail chalones which can have an effective role in the spermatogenesis process (18). 

In the present study, the protective effect of L-carnitine and testis-homogenized tissue following busulfan treatment is hypothesized. Stereological technique was used for estimation volumes of testis and interstitial tissue, volume, length and diameter of seminiferous tubules and germinal epithelium height. Stereological method is an extremely efficient and widely used method in the medical field.

## Materials and methods

**Ethics**

All animal experimentation protocols were carried out under supervision of the Ethics Committee of Shiraz University of Medical Sciences.

**Animals**

In this experimental study, twenty adult male Spraque-Dawley rats (about 180±20 gr) were selected from the laboratory animal center of Shiraz University of Medical Sciences. Rats were maintained under standard conditions (12 hr dark/night and free access to food and tap water throughout the experiment).

**Drugs preparation**

Preparation of busulfan solution: Busulfan (Sigma, B2635, USA) was first dissolved in DMSO (dimethyl sulfoxide), (Sigma, D2650, USA), then an equal volume of sterile water was added to obtain a final busulfan concentration of 5 mg/ml. Preparation of L-carnetine: 200 mg L-carnitine (Sigma, C0283-5G, USA) was dissolved in 10 ml of distilled water to obtain a concentration of 20 mg/ ml.

**Experimental design**

Rats were randomly divided into four different experimental groups:

Group I (Control) received a single dose of DMSO (I.P.) plus daily I.P. injection of 1mL of distilled water for 48 days. 

Group II (Bus) received a single I.P. injection of busulfan (Bus) at a dose of 10 mg/kg plus daily I.P. injection of 1mL of the distilled water. 

Group III (Bus+THT) received a single I.P. injection of busulfan plus 1mL of testis-homogenized tissue (THT) daily by oral gavages.

Group IV (Bus+L-car) received a single I.P. injection of busulfan plus 100 mg/kg/day I.P. injection of L-carnitine (L-car). Administration of L-carnitine and THT started 1day after injection of busulfan and was continued for 48 days (according to the spermatogenesis period in rats) (19).

At the end of the treatment period, the rats were weighed and anesthetized using diethyl ether, then killed; the testes was fixed by perfusion with 4% formaldehyde in buffered solution for 20 minutes and afterwards their left testes were taken out and weighed. The volume of testis was estimated using Archimedes principle, by immersion in distilled water (20) and then testicular tissues were immediately fixed in 4% formaldehyde in buffered solution for an additional 72 hours for histological and stereological study. 

**Hormone assays**

Before perfusion, in order to measure the plasma testosterone and estradiol levels, blood samples were immediately collected by cardiac puncture and the plasma separated from the blood cells by centrifugation (2500 rpm for 30 mins). All blood samples were then immediately stored at -20^o^C until further analyses. Testosterone and estradiol levels in plasma of rats were measured by Radio Immuno Assay, using a commercial kit (RIA KIT, IMMUNOTECH, Czech Republic). The testosterone kit's sensitivity was 0.025ng/ml and the esteradiol kit, 6 pg/ml. 

**Sperm quality**

The sperm samples were obtained from the distal region (1.0 cm) of the left vas deferens, placed in a plate containing 2cc of Hank’s Balanced Salt Solution (HBSS) and agitated gently at 37ºC for 3 min.

**Sperm count**

Specimens were spread on a hemocytometer and the heads were counted manually under a light microscope. Data were expressed as total number of sperm/ml (21).

$\mathrm{Spermcount}=\frac{\mathrm{No. of spermatozoa a counted}\times \mathrm{Dilution factor}\times \mathrm{Depth factor}}{\mathrm{No. of areas counted}}$

**Sperm morphology**

Spermatozoa were classified as normal or abnormal. Abnormality was classified into a variety of head and tail abnormalities, such as blunt hook, banana-head, amorphous, pin-head, two-head, two-tail, small head and bent tail. The sperm smears were stained with Eosin Y and evaluated in 10 microscopic fields and 200-300 sperm per animal were then analyzed. Finally, this parameter (normal morphology sperm fraction) was defined as the mean number of normal sperm × 100 /total number of sperm (21).

**Stereological analysis of testis**

**Systematically random sampling in isotropic uniform random sections**

**Figure 1:**
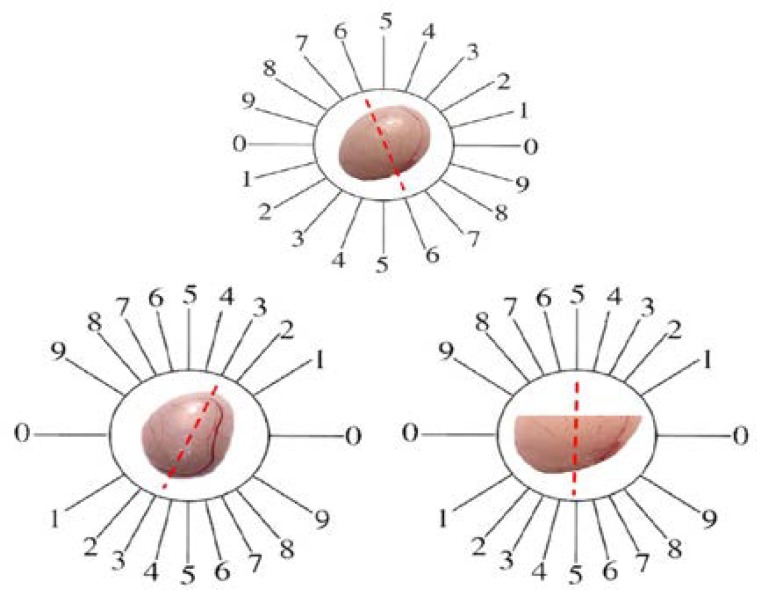
Orientator method, to achieve the isotropic uniform random sections. (Top) The whole testis is placed on Φ Clock . A random number between 0 and 10 is selected (here 6), and the testis is sectioned into two halves, with a blade at that direction. (Bottom) The cut surface of each half of the testis is then placed on the 0-0 direction of θ Clock and the second cuts are done (here 3 and 5).

The orientator method was used to obtain Isotropic Uniform Random (IUR) sections. For this purpose, testis was randomly placed on the φ clock (Figure 1). After choosing a random number from one to nine, an appropriate cut was made along the selected number, which resulted in two pieces of testis. The first piece was then placed on the θ clock along with its previous cut surface on the 0-0 axis, then a random number was selected again and a parallel cut was made along the selected number. The other piece that resulted from the cut made on the φ clock was placed on the θ clock vertically so that its cut surface overlapped the 0-0 axis (Figure 1). 

This piece was also cut parallel along a randomly selected number; eight to ten slabs were collected from the tissue after applying orientator method. The slabs were then embedded in paraffin and sections of 5 and 16-μm thickness were cut using the microtome and after tissue processing, stained using Heidenhain’s Azan trichrome and H&E method.

**Estimating the shrinkage and the total volume of testis**

**Figure 2:**
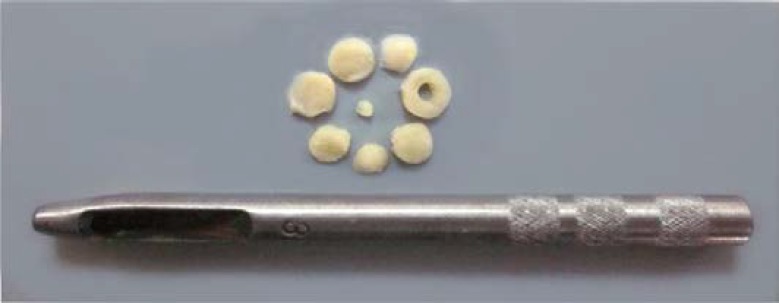
Trocar and IUR sections.

To estimate the shrinkage, a circle was punched from a testis slab using a trocar (Figure 2). The vertical diameters and area of the circular piece of the testis were measured and their mean radius was estimated and considered as the pre fixing radius (r_before_). The cut surfaces of the slabs and circular piece were embedded in paraffin block. After sectioning and staining each slide, the mean radius of the circular piece was considered as the post fixing radius (r_after_). Using the following equation, the amount of shrinkage in each testis was estimated.

$\mathrm{volume shrinkage}=1-{\left(\frac{{r_{\mathrm{after}}}^{2}}{{r_{\mathrm{before}}}^{2}}\right)}^{1.5}$

To obtain the true volume of testis, the amount of shrinkage was subtracted from the volume estimated by the immersion method.

$V_{\mathrm{finaltestis}}=V_{\mathrm{primary}}\times (1-\mathrm{volumeshrinkage})$

**Stereological analysis of testis**

Microscopic analyses were done using a video-microscopy system made up of a microscope (E-200, Nikon, Japan) linked to a video camera (CCD, Hyper HAD (, a computer and a flat monitor (Platrun LG). To estimate each parameter, 10-14 microscopic fields were examined in each testis. Microscopic fields were selected by systematic random sampling. 

Briefly, the slide was moved at equal intervals along the X- and Y-axis using a stage micrometer. By means of the stereology software designed at our laboratory (Morphometry & Stereology Research Centre, Shiraz University of Medical Sciences, Shiraz, Iran) relevant grids (test probes) were over-laid on the monitor.

**Estimation of volume of the tubules and interstitial tissue**

To estimate the total volume of the seminiferous tubules and interstitial tissue, 5 µm sections were used. A grid of points was overlaid on the monitor image of the testis (Figure 4). The volume density “Vv (structure/testis)” of the tubules or interstitial tissue was estimated using point counting at final magnification of 160 and the following formula: 

$V_{v_{\left(\frac{\mathrm{structure}}{\mathrm{testis}}\right)}}=\frac{\sum P_{\mathrm{structure}}}{\sum P_{\mathrm{total}}}$

Where the “∑P_Structure_” was the number of points hitting the profiles of the tubules or interstitial tissue and “∑P_Total_” was the number of points hitting the testis. The total (absolute) volume was obtained by multiplying the density by the final testis volume:

**Estimation of the length and diameter of the tubules**

The length density of the seminiferous tubules was estimated by overlaying an unbiased counting frame randomly on the monitor at final magnification of 160 (Figure 5). The length density (L_V_) of the tubule was calculated as: 

$L_{v}=\frac{2\times \sum Q}{(\frac{a}{f})\times \sum f}$

Where “∑Q” was the total number of the tubule profiles counted per rat testis, (a/f) was the area of the counting frame (625 µm×625 µm) and “∑f” is the total number of frames counted in each animal. The total length of the tubule “L” was calculated by multiplying the length density (L_V_) by the total volume of the tubules.

$L=L_{v}\times V_{\mathrm{final}}$

The diameter of the tubules was measured on the tubules are selected at random by using the upper right corner of the unbiased counting frame applied for estimating the length of the tubules. The diameter was measured perpendicular to the long axis where the tubule was widest (Figure 5). The minimum diameter was used as it was not affected by possible obliquity of the section and was the most reliable measurement of this parameter.

**Estimation of the height of epithelium of the tubules**

To estimate the height of the germinal epithelium, on the 5 µm thick sections at final magnification of 460, the following equation was used:

$H=\frac{V_{v}}{S_{v}}$

In which “Vv” and “Sv” were the volume density and surface density of the germinal epithelium, respectively. The volume density (Vv) of the germinal epithelium was obtained by point counting method. To estimate the surface density (Sv) of the germinal epithelium, a linear test probe was used (Figure 6). The total number of points was superimposed on the germinal epithelium (∑p), and the length of each line (l/p) and the number of intersections of the linear test probe with the inner surface of the germinal epithelium (∑I) were recalculated. The surface density (Sv) was then estimated using the following equation:

$S_{v}=\frac{2\times \sum I}{\sum \mathrm{p.1}/p}$

**Estimating the germinal epithelium cells number**

To estimate the total number of Leydig cells, sertoli cells, spermatogonia, spermatocytes, and round spermatids, the 16 µm thickness sections and a high numerical aperture (NA=1.4) oil immersion lens were used. By means of the stereology software, an unbiased counting frame was superimposed on the images of the testis sections viewed on the monitor. 

The number of cells was estimated using “optical dissector” method. The frame avoids the “edge effect” and biased counting of the particles, and all the nuclei profiles regardless of their shape or size are counted by the frame and have the same probability of being sampled. The optical section was moved downwards in z-axis. To have an unbiased counting, a guard zone was considered for estimation i.e., the first 5 µm was ignored. 

This was achieved using a microcator (HeidenhainMT-12, Germany) that measures the z-axis traveling (depth). Any cell whose nucleus was in focus at the first 5 µ m plane was ignored. Any nuclear profile within the next traveling 6 µm optical section (height of dissector) was selected if it lay in the counting frame or touched the inclusion borders and did not touch the exclusion borders of the frame (Figure 7). The numerical density (Nv), or number of cells in the unit volume of the germinal epithelium was estimated using:

$N_{v}=\frac{\sum Q}{\sum A}\times h$

where “∑Q” was the number of nuclei coming into focus in the dissector height, “∑A” was the total area of the unbiased counting frame in all microscopic fields and “h” was the height of dissector (6 µm here). The total number of nuclei was estimated by multiplying the numerical density (Nv) by the total volume of the epithelium.

$N=N_{V}\times V_{\mathrm{final}}$

**Length of sperm flagella**

The spermatozoon samples prepared for morphology assessment were used for stereological estimating. A ×60 oil immersion objective with a numerical aperture of 1.4 was used to achieve a better recognition of the tail. The length was estimated according to the stereological methods for estimating the length in two-dimensional space. The microscopic fields were sampled according to the rules for systematic random sampling. Briefly, the microscope stage was moved in an equal interval along the X- and Y-direction of the microscope stage, using the stage micrometer of the microscope. Between 100 and 150 spermatozoa were sampled on each slide. 

To achieve an acceptable precision it has been advised that at least 5 specimens in each group undergo the analysis and a 100-200 probe interaction (e.g. sampling of 100-200 spermatozoon heads by counting frame, or 100-200 intersections of Merz grid with spermatozoon tails) should be considered. In each sampled microscopic field, a test system including two elements was superposed on the image on the monitor. The first component was the unbiased counting frame. 

In this frame, if a spermatozoon's head lay inside the frame and did not touch the forbidden lines (left and inferior borders of the frame), it was sampled. Merz grid is a curve consisting of two equal semicircles (Fig.8). The following formula was used for estimating the mean spermatozoon tail length:

$l=\frac{\pi }{2}\times \frac{a}{1}\times \frac{1}{\mathrm{asf}}\times \sum I$

$L=\frac{l}{\sum n}$

Where "a/1" was the Merz grid constant obtained dividing the area of each basic tile by the length of the semicircles. Within this tile there were two semicircles of length π.d., (simple formula for estimation of the perimeter of a circle), where "d" was the diameter of the circle and "asf" was the area sampling fraction. The "asf" was computed by dividing the area of the rectangle by the area of the unbiased counting frame. "ΣI" was the total intersections of the tails (mid piece or total tail) with the semicircles (Figure 8). "ΣN" was the total number of the counted spermatozoa in the unbiased counting frame in all fields.

**Statistical analysis**

For statistical analysis, One-way Anova test was used with LSD post Hoc. P-values less than 0.05 were considered as statistically significant. Statistical analysis was performed using SPSS 17. Relevant plots were drawn with Microsoft office excel 2007.

## Results

**Body and testis weight and testis volume**

The mean body weight in the third experimental group was significantly less than that of the first and second experimental groups and the control group (p<0.006). Testis weight and volume was reduced in the first, second and third experimental groups in comparison with the control group (p<0.0001), but testis weight and volume increased after treatment of rats with testis-homogenized tissue, in comparison with the first and third experimental groups (p<0.0001). There was no significant difference in these parameters between the first and third experimental groups (Table I).

**Tubule and interstitial tissue volume**

The data show that tubule volume in animals treated with the busulfan, busulfan + L-carnitine and busulfan+ testis-homogenized tissue decreased in comparison with the control group (p<0.0001). It is also obvious that the animals receiving busulfan+ testis-homogenized tissue showed significant increase compared with those that received busulfan and busulfan+ L-carnitine (p<0.003). 

There was no significant difference between the first and third experimental groups (Table I). Total epithelial volume showed no significant differences between the first experimental and control groups, but this parameter in the first and third experimental group has decreased in comparison with the control group (p<0.02). Total epithelial volume in animals treated with busulfan+testis-homogenized tissue significantly increased in comparison with busulfan, and busulfan+ L-carnitine (p<0.005) (Table I).

**Tubule length** **and diameter and** **germinal** **epithelium height**

No significant differences were seen in tubule length in any of the groups. The tubular diameter and the height of germinal epithelium were measured and, as compared with the control group, both values of stereologic parameters were statistically significantly reduced in the animals treated with busulfan, busulfan+L-carnitine (p<0.002). But testis-homogenized tissue treatment significantly increased the tubule diameter and germinal epithelium height compared to the first and third experimental groups (p<0.01).

**Number of the different cells of germinal epithelium**

The total number of sertoli and Leydige cells did not show any significant difference in any of the groups (Table III). According to the results, the total number of spermatogonia, primary spermatocyte and round spermatid cells in animals treated with the busulfan, and busulfan+L-carnitine significantly decreased in comparison with the control group (p<0.007). Testis-homogenized tissue treatment significantly increased the total number of spermatogonia, primary spermatocyte and round spermatid cells compared to the first and third experimental groups (p<0.003). 

The total number of primary spermatocyte in the third experimental group significantly increased in comparison with the first experimental group (p<0.01). As compared to the control group, examination of the testis-homogenized tissue revealed a significant decrease in the total number of spermatogonia (p<0.01) (Table III).

**Total sperm count, normal morphology sperm** **% and Length of sperm flagella**

The data reveal that total sperm count, as well as normal morphology sperm percentage was reduced significantly in the busulfan treated animals in comparison with the other groups (p<0.05). However, these parameters did not show any significant differences in the other groups (Table IV).

**Hormone assays**

The testosterone and estradiol levels in plasma of rats did not show any significant difference in any of the groups (Table V).

**Table I:** Mean and total observed variation (CV) of the body and testis weight (g), testis volume (mm3) and total volume of the semniferous tubules and interstitial tissue (mm3) in the control, busulfan, busulfan + testis homogenized tissue (THT) and busulfan + L-Carnitine (L- Car) treated groups

**Groups**		**Body weight**	**Testis weight**	**Testis volume**	**Total volume of the tubules**	**Total volume of the ** **Interstitial tissue**
Control						
	Means	298	1.53*	1477*	858*	329****
	CV	0.05	0.1	0.1	0.14	0.38
Busulfan						
	Means	303	0.8	764	441	241
	CV	0.06	0.12	0.13	0.15	0.11
Busulfan + THT						
	Means	287	1.21***	1089***	625***	372***
	CV	0.11	0.04	0.04	0.02	0.12
Busulfan + L- Car						
	Means	271**	0.83	817	463	234
	CV	0.05	0.12	0.12	0.18	0.26

* p< 0.0001, Control vs. other groups.

** p< 0.006, Busulfan + L-car vs. busulfan and control.

*** p<0.005, Busulfan + THT vs. busulfan and busulfan + L- Car.

**** p<0.02 Control vs. busulfan and busulfan + L- Car.

**Table II:** Mean and total observed variation (CV) of the semniferous tubules length (M) and diameter (µm) and germinal epithelium height (µm) in the control, busulfan, busulfan + busulfan + THT and busulfan + L- Car treated groups

**Groups**		**Tubule length (M)**	**Tubule diameter (µm)**	**Germinal epithelium height ** **(µm)**
Control				
	Means	19.0	265*	119**
	CV	0.26	0.12	0.16
Busulfan				
	Means	16.7	186	57
	CV	0.26	0.10	0.23
Busulfan + THT				
	Means	19	223***	102***
	CV	0.10	0.09	0.14
Busulfan + L- Car				
	Means	15	190	58
	CV	0.20	0.07	0.31

* p< 0.002, Control vs. other groups.

** p<0.0001, Control vs. busulfan and busulfan + L- Car.

*** p<0.01, busulfan + THT vs. busulfan and busulfan + L- Car.

**Table III:** Mean and total observed variation (CV) of the number (×10^6^) of the different cells of germinal epithelium in the control, busulfan, busulfan +THT and busulfan + L- Car treated groups

**Groups**		**Sertoli Cell**	**Spermatogonia**	**Primary spermatocyte**	**Round spermatid**	**Leydige cell**
Control						
	Means	28.4	9.8*	124.8**	319.2**	21.9
	CV	0.1	0.10	0.04	0.09	0.06
Busulfan						
	Means	27.5	8.0	84.9	238.0	21.1
	CV	0.18	0.10	0.11	0.146	0.06
Busulfan + THT						
	Means	29.2	8.7	120.4***	305.2***	20.9
	CV	0.11	0.08	0.02	0.14	0.04
Busulfan + L- Car						
	Means	27.9	8.6	94.1****	238.6	20.7
	CV	0.16	0.08	0.10	0.15	0.05

* p< 0.01, Control vs. other groups

** p<0.007, Control vs. busulfan and busulfan + L- Car

*** p<0.003, Busulfan + THT vs. busulfan and busulfan + L- Car

**** p<0.01, Busulfan + L- car vs. busulfan

**Table IV:** Mean±SD of the sperm count, percentage of normal morphology sperm and length of flagella in the control, busulfan, busulfan + THT and busulfan + L- Car treated groups

**Groups**	**Sperm count (×10** ^6^ ** /mL)**	**Normal Morphology sperm ** **(%)**	**Length of flagella** ** (µm)**
Control	58.6±2.53	83±6.36	135.4±13.4
Busulfan	32.4±2.88*	64±26.24*	133.2±21.2
Busulfan + THT	54.8±2.56	89±6.67	156.3±52.8
Busulfan + L- Car	40.8±2.56	88±21.26	174.4±43.5**

* p< 0.05, Busulfan vs. other groups

** p< 0.01, Busulfan + L- Car vs. busulfan and control groups

**Table V:** Mean and total observed variation (CV) of the estradiol and testosterone hormones in the control, busulfan, busulfan + THT and busulfan + L- Car treated groups

**Groups**		**Estradiol hormone (pg/ml)**	**Testosterone hormone (ng/ml)**
Control			
	Means	155.1	1.2
	CV	0.25	0.83
Busulfan			
	Means	157.6	1.2
	CV	0.23	0.87
Busulfan + THT			
	Means	139.0	1.4
	CV	0.03	0.62
Busulfan + L- Car			
	Means	160.1	1.4
	CV	0.19	0.73

**Figure 3:**
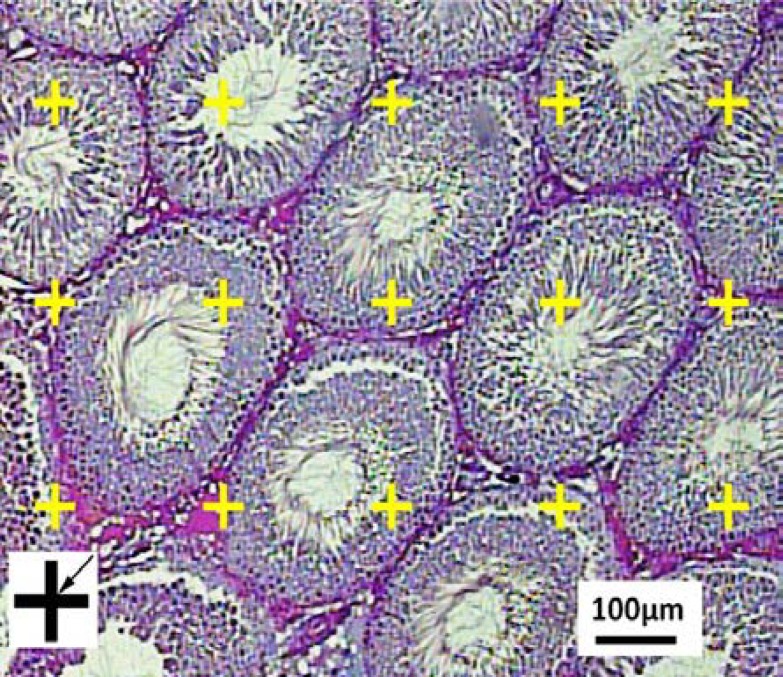
Volume density estimation using point counting. The total number of points hitting the interstitial tissue, lumen, or epithelium is counted and divided by the total number of points hitting the reference space (here testis). At the corner of this figure, a point is presented. The right upper corner of the cross is considered as the point (arrow), and it is counted only if the right upper corner hits the tissue.

**Figure 4:**
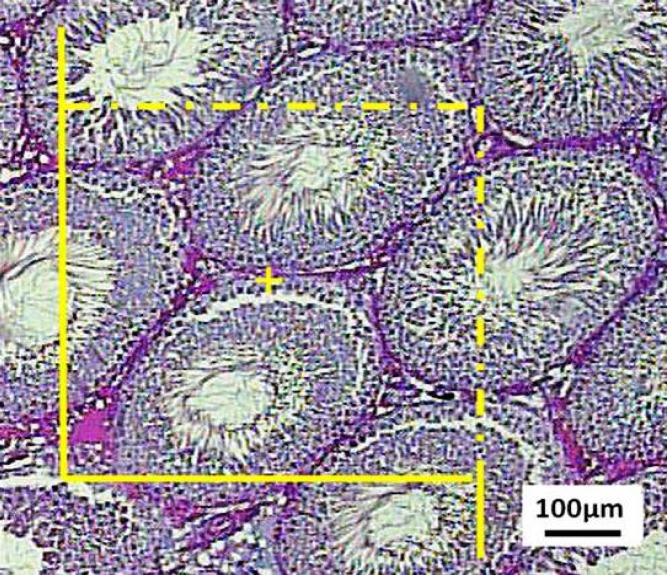
The length density of the seminiferous tubules is estimated by an unbiased counting frame on the images. The tubule profiles are completely inside the counting frame or partly inside the counting frame but only those touching the top and right lines are counted

**Figure 5:**
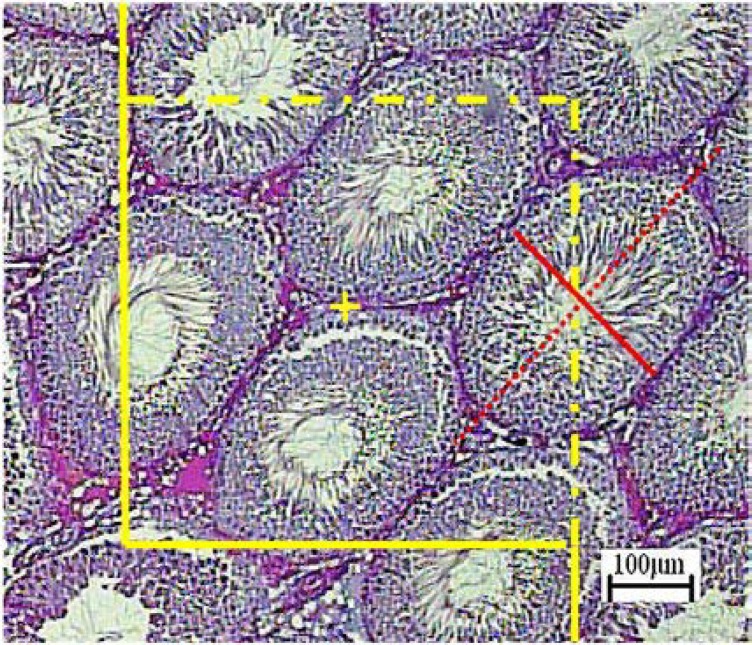
The diameter of the tubules is measured on the tubules sampled by the counting frame. The diameter is measured perpendicular to the long axis where the tubule is widest

**Figure 6:**
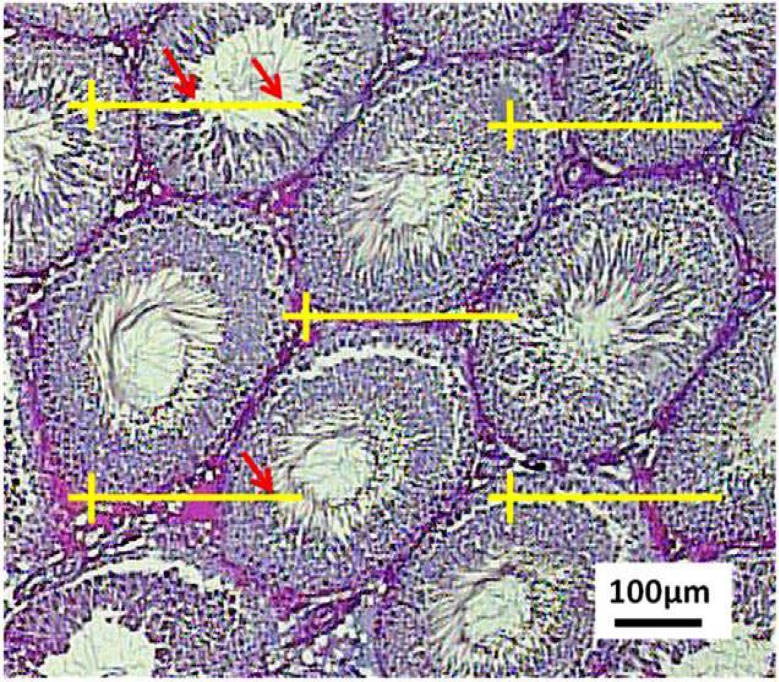
To estimate the height of the germinal epithelium, the volume density is divided by surface density of the germinal epithelium. To obtain the volume density, a point grid superimposes on each image of the testis and to estimate the surface density (Sv).

**Figure 7:**
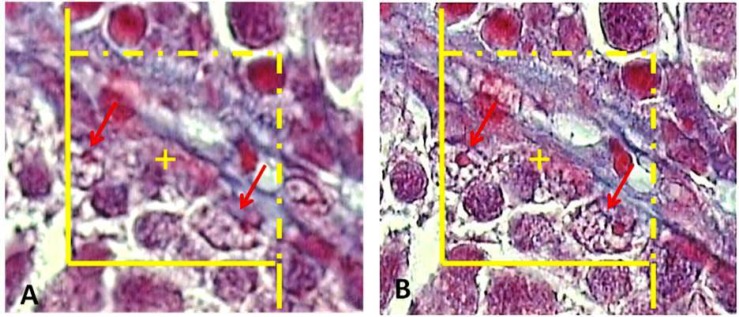
To estimate the total number of the cells (here sertoli cells) using the optical disector method, an unbiased counting frame is superimposed on the images (A). Any nuclei of sertoli cells that come into maximal focus within the traveling optical section

**Figure 8:**
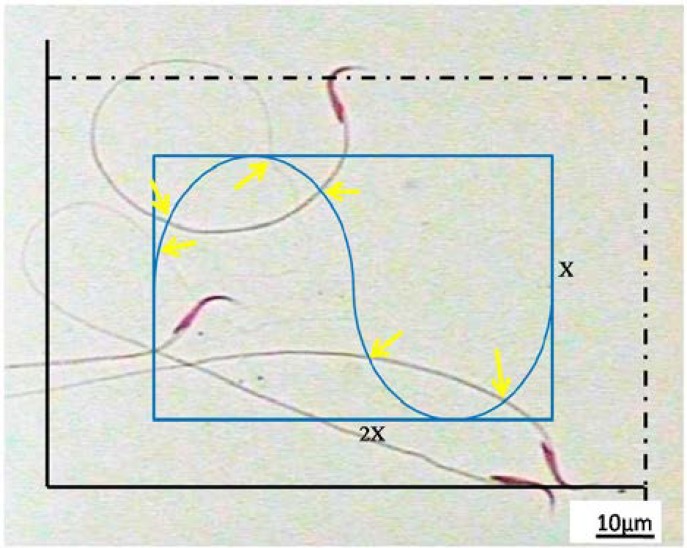
This figure shows the spermatozoa heads and tails in a microscopic field. To estimate the tail length, a test system consisting of two components was superimposed on the image. The first was an unbiased counting frame (the large frame) with acceptance (dotted) and forbidden (bold) lines. If the spermatozoa head lie inside the frame and did not touch the exclusion lines, they were sampled (here three spermatozoa). Another component was a rectangle with a Merz grid inside it (the curve with two semicircles). The arrow heads show the intersections between the Merz grid and the tails.

## Discussion

The administration of busulfan to male patients with malignant diseases may cause temporary or permanent sterility (1). Busulfan induced azoospermia and normal process' disruption of spermatogenesis (22). Estimation of testis parameters such as testis volume and number of germinal cells by stereological methods lead to better evaluation of the spermatogenesis process.

In this study, busulfan decreases sperm count and total number of spermatogonia, primary spermatocyte and spermatid. Zhengwei *et al* showed that there was a direct relationship between testis volume and germinal cells number in primates (23). In this study, testis weight, volumes of seminiferous tubules and testis, germinal epithelium height and seminiferous tubule diameter significantly decreased in the animals treated by busulfan. Researches show that busulfan produces free radicals that directly affect DNA or the destruction of the genome. Bucci *et al* showed that busulfan caused chromosomal abnormalities and dominant lethal mutations in sperm (2).

Therefore, it seems busulfan inhibit the spermatogenesis process, especially by oxidative damage. Other mechanism suggested that busulfan increased the level of ck-18, a surface marker on sertoli cell. The elevation of this marker caused spermatogenesis disorder and infertility (24). This study also showed that busulfan increased sperm abnormality and reduced flagella length. The tail of a spermatozoon acts in the swimming ability of the cell and consequent fertilization. It is likely that busulfan reduced sperm motility by reducing flagella length. 

Our investigation revealed that busulfan had no effect on the number of sertoli and Leydige cells. In this project, busulfan did not affect the testosterone and estradiol levels. Although Howel *et al* reported that busulfan increased the level of LH that induce differentiation of Leydige cells, Aich and Vecino showed that busulfan caused the irregular arrangement of the sertoli cells but did not affect the number of these cells (3 , 25). It seems these changes are relative to the level of surface marker on the sertoli in the response by busulfan (26). These results show that busulfan induces testicular injury. Therefore, a research on material or drugs that can reduce busulfan's side effects is necessary.

In the present study, administration of L-carnitine and THT in busulfan treated animals prevent gonadal toxicity. Based on this research, the length of sperm flagella and sperm count significantly increased in animals treated with L-carnitine in comparison with the busulfan-treated group. Lenzi believed that L-carnitine affected the sperm quality by its positive effect on the epididymal environment, that lead to reduced phagocytosis of gametes and, therefore, increased sperm count (27). Other studies have suggested that L-carnitine improved sperm motility and chromatin quality via antioxidant properties and the enhanced glucose uptake by sperm (14, 28). 

But the other stereological parameters such as volume of seminiferouse tubules and germinal epithelium height did not show any significant difference between either the busulfan or busulfan+ L-carnitine treated animals. It seemed these findings are due to duration of injection or the lower levels of the L-carnitine in the testis compared with its level in the epididymis. In this study, THT is more effective than L-carnitine in reducing some of the side effects of busulfan on the testis. THT increased testis volume (6.5%) and weight (8.6%), tubule and interstitial tissue volume (6.9% and 11.7% respectively), seminiferous tubule diameter (3.8%), germinal epithelium height (13%) and sperm count (7.5%), and decreased sperm with abnormal morphology (1%) in comparison with the L-carnitine+ busulfan treated group.

It seems the material of the THT causes positive effect on the testis parameters. The previous studies showed that spermatognial chalones, as a mitotic inhibitory factor in the testis extract, can inhibit proliferation of A spermatogonia but has no effect on the B spermatogonia and other germinal cells (29, 30). However, the molecular mechanism of action by THT and how it affects male fertility has not been completely clarified and more investigation is needed.

## Conclusion

In conclusion, it seems that the use of L-carnitine and THT with busulfan decreases some side effects of this drug on the male reproductive system. However, in our study, THT was more effective than L-carnitine and led to the recovery of both testis and sperm parameters after treatment with busulfan. 

## References

[1] Bishop JB, Wassom JS (1986). Toxicological review of busulfan (Myleran). Mutat Res.

[2] Bucci LR, Meistrich ML (1987). Effects of busulfan on murine spermatogenesis: cytotoxicity, sterility, sperm abnormalities, and dominant lethal mutations. Mutat Res.

[3] Howell SJ, Shalet SM (2001). Testicular function following chemotherapy. Hum Reprod Update.

[4] Howell SJ, Radford JA, Ryder WD, Shalet SM (1999). Testicular function after cytotoxic chemotherapy: evidence of Leydig cell insufficiency. J Clin Oncol.

[5] Meistrich ML (2009). Male gonadal toxicity. Pediatr Blood Cancer.

[6] Mohammadghasemi F, Faghani M, Falah Karkan M (2010). The protective effect of melatonin on sperm parameters, epididymis and seminal vesicle morphology in adult mouse treated with busulfan. J Iran Anat Sci.

[7] Perez-Crespo M, Pericuesta E, Perez-Cerezales S, Arenas MI, Lobo MV, Diaz-Gil JJ (2011). Effect of liver growth factor on both testicular regeneration and recovery of spermatogenesis in busulfan-treated mice. Reprod Biol Endocrinol.

[8] Agarwal A, Said TM (2004). Carnitines and male infertility. Reprod Biomed Online.

[9] Bremer J (1983). Carnitine-metabolism and function. Physiol Res.

[10] Izgut-Uysal VN, Agac A, Derin N (2003). Effect of L-carnitine on carrageenan-induced inflammation in aged rats. Gerontology.

[11] Ferrari R, Merli E, Cicchitelli G, Mele D, Fucili A, Ceconi C (2004). Therapeutic effects of L-carnitine and ropionyl-L-carnitine on cardiovascular diseases: a review. Ann N Y Acad Sci.

[12] Onem G, Aral E, Enli Y, Oguz EO, Coskun E, Aybek H (2006). Neuroprotective effects of L-carnitine and vitamin E alone or in combination against. J Surg Res.

[13] Boerrigter ME, Franceschi C, Arrigoni-Martelli E, Wei JY, Vijg J (1993). The effect of L-carnitine and acetyl-L-carnitine on the disappearance of DNA single-strand breaks in human peripheral blood lymphocytes. Carsinogenesis.

[14] Moretti S, Famularo G, Marcellini S, Boschini A, Santini G, Trinchieri V (2002). L-carnitine reduces lymhocyte apoptosis and oxidant stres in HIV-1-infected subjects treated with zidovudine and didanosine. Antioxid Redox Signal.

[15] Khademi A, Safdarian L, Alleyassin A, Agha-Hosseini M, Akbari Hamed E, Saeidi Saeidabadi H (2004). The effect of L-carnitine on sperm parameters in patients candidated for Intracytoplasmic Sperm Injection. Iran J Reprod Med.

[16] Grollman A, Williams JR, Harrison TR (1942). Effects of renal extract on hypertension. Bull N Y Acad Med.

[17] Tanzer RC (1932). The Effect of Testicle Extract on the Growth of Transplantable Mouse Tumors. J Exp Med.

[18] De Rooij DG, Van Dissel-Emiliani FM, Van Pelt AM (1989). Regulation of spermatogonial proliferation. Ann N Y Acad Sci.

[19] Saki G, Rahim F, Alizadeh K (2009). Effect of forced swimming stress on count, motility and fertilization capacity of the sperm in adult rats. J Hum Reprod Sci.

[20] Elias H, Hyde DM (1980). An elementary introduction to stereology (quantitative microscopy). Am J Anat.

[21] Seed J, Chapin RE, Clegg ED, Dostal LA, Foote RH, Hurtt ME (1996). Methods for assessing sperm motility, morphology, and counts in the rat, rabbit, and dog: a consensus report. ILSI Risk Science Institute Expert Working Group on Sperm Evaluation. Reprod Toxicol.

[22] Anjamrooz SH, Movahedin M, Mowla SJ, Bairanvand SP (2007). Assessment of morphological and functional changes in the mouse testis and epididymal sperms following busulfan treatment. Iran Biomed J.

[23] Zhengwei Y, McLachlan RI, Bremner WJ, Wreford NG (1997). Quantitative (stereological) study of the normal spermatogenesis in the adult monkey (Macaca fascicularis). J Androl.

[24] Bar-Shira Maymon B, Yogev L, Marks A, Hauser R, Botchan A, Yavetz H (2004). Sertoli cell inactivation by cytotoxic damage to the human testis after cancer chemotherapy. Fertil Steril.

[25] Vecino P, Uranga JA, Arechaga J (2001). Suppression of spermatogenesis for cell transplantation in adult mice. Protoplasma.

[26] Udagawa K, Ogawa T, Watanabe T, Yumura Y, Takeda M, Hosaka M (2001). GnRH analog, leuprorelin acetate, promotes regeneration of rat spermatogenesis after severe chemical damage. Int J Urol.

[27] Lenzi A, Sgro P, Salacone P, Paoli D, Gilio B, Lombardo F (2004). A placebo-controlled double-blind randomized trial of the use of combined l-carnitine and l-acetyl-carnitine treatment in men with asthenozoospermia. Fertil Steril.

[28] Aliabadi E, Soleimani Mehranjani M, Borzoei Z, Talaei-Khozani T, Mirkhani H, Tabes H (2012). Effects of L-carnitine and L-acetyl-carnitine on testicular sperm motility and chromatin quality. Iran J Reprod Med.

[29] Hochereau-de-Reviers MT, Viguier-Martinez MC, Perreau C (1982). Effects of Supplementation with Impuberal or Adult Testicular Protein Extracts on Genital Tract and Testicular Histology as well as Hormonal Levels in Adult Busulfan Treated Rats. Andrologia.

[30] Irons MJ, Clermont Y (1979). Spermatogonial chalone(s): effect on the phases of the cell cycle of type A spermatogonia in the rat. Cell Proliferation.

